# Characteristics of Polysilicon Wire Glucose Sensors with a Surface Modified by Silica Nanoparticles/γ-APTES Nanocomposite

**DOI:** 10.3390/s110302796

**Published:** 2011-03-02

**Authors:** Jing-Jenn Lin, Po-Yen Hsu, You-Lin Wu, Jheng-Jia Jhuang

**Affiliations:** 1 Department of Applied Materials and Optoelectronic Engineering, National Chi Nan University, Puli, Nantou 54561, Taiwan; 2 Department of Electrical Engineering, National Chi Nan University, Puli, Nantou 54561, Taiwan; E-Mails: s95323902@ncnu.edu.tw (P.-Y.H.); ylwu@ncnu.edu.tw (Y.-L.W.); s98323508@ncnu.edu.tw (J.-J.J.)

**Keywords:** line width dependence, polysilicon wire, γ-APTES, PDMS, silica nanoparticles

## Abstract

This report investigates the sensing characteristics of polysilicon wire (PSW) glucose biosensors, including thickness characteristics and line-width effects on detection limits, linear range and interference immunity with membranes coated by micropipette/spin-coating and focus-ion-beam (FIB) processed capillary atomic-force-microscopy (C-AFM) tip scan/coating methods. The PSW surface was modified with a mixture of 3-aminopropyl-triethoxysilane (γ-APTES) and polydimethylsiloxane (PDMS)-treated hydrophobic fumed silica nanoparticles (NPs). We found that the thickness of the γ-APTES+NPs nonocomposite could be controlled well at about 22 nm with small relative standard deviation (RSD) with repeated C-AFM tip scan/coatings. The detection limit increased and linear range decreased with the line width of the PSW through the tip-coating process. Interestingly, the interference immunity ability improves as the line width increases. For a 500 nm-wide PSW, the percentage changes of the channel current density changes (ΔJ) caused by acetaminophen (AP) can be kept below 3.5% at an ultra-high AP-to-glucose concentration ratio of 600:1. Simulation results showed that the line width dependence of interference immunity was strongly correlated with the channel electrical field of the PSW biosensor.

## 1. Introduction

As any subtle biological or chemical change in the human body may affect the performance of living systems, the development of high-sensitivity biosensors to detect low concentrations of molecules such as DNA, proteins, *etc.*, has been a high-profile effort in recent years. Since 2001, when Leiber’s research team used silicon nanowire (SiNW) to develop a nano-biosensor, many studies have pointed out that this one-dimensional structure has the potential to serve as the foundation for a new generation of nanotechnology biosensors [1–5]. This is because this type of structure allows for a highly sensitive and simple detection method, resulting in SiNW being successfully deployed in chemical, biomedical and physiological signal research. Previous studies have shown SiNW to be useful as a sensing channel for detecting proteins, viruses and the molar range to tens of femto pH solutions. Thus future developments might allow for nanostructure-based biosensors being applied to single molecule detection and micro-system components used for analyzing a variety of molecules. However, because the current vapor-liquid-solid (VLS) fabricating methods for SiNW cannot effectively control the performance difference between components, there are still many problems which need to be overcome, including fabrication methods for routinely fabricating low cost scalable biosensors, and reproducibility [6]. Of particular importance is the development of an even sensing film coating on the sensor surface, which will determine whether the technology is successful in creating highly reproducible biosensors. In our previous study, we proposed a polysilicon material as the wire used in the detection of glucose and DNA. This approach is less expensive and more compatible with advanced semiconductor fabrication. More importantly, this structure serves as the foundation for biochemical sensors simultaneously offering high sensitivity, high immunity to interference and dry-type detection [7–10]. In addition, our FIB processed C-AFM tip has also been proven to be effective in coating very small amounts of solution to the device surface. We believe this technology can effectively help in the fabrication of very small and highly reproducible nano-semiconductor biosensors [8,10].

*In vitro* glucose level monitoring is important in clinical diagnostics for patients with diabetes mellitus, and there is a significant need for a highly sensitive and reliable glucose biosensor. Various methodologies for glucose detection have been reported, including amperometric, potentiometric and impedimetric applications [10–13]. Sensitivity, linear working range, detection limits, interference resistance and stability play main roles for the glucose sensor investigation under physiological condition detection [14–16]. In our previous work, we used the FIB processed C-AFM tip to apply γ-APTES+NPs+UV as the sensing layer coated onto the PSW surface to produce a very-low concentration, highly sensitive, highly selective and durable glucose detector with a wide linear range [8,10]. We believe the development of polysilicon wires could serve as a very appropriate foundation for highly sensitive and highly reproducible glucose sensors.

In this paper, we focus on the effect of different line widths on the sensitivity and selectivity of the detection capabilities of the PSW glucose sensor. We compare film thicknesses produced by spin coating and FIB processed C-AFM tip coating, and the characteristics of different widths between the PSWs, and show the FIB processed C-AFM tip coating method to be superior. Electroactive interference substances may be decomposed by different electric field strengths and thus affect the sensing properties of the PSW glucose sensor [17–19]. Therefore, to better understand the effect of different PSW sensor line widths and electric field distribution on interference immunity ability, we also use the MATLAB numerical analysis software to simulate the Poisson’s equation to understand the electric field distribution of different PSW channel widths. We will demonstrate that the strength of the PSW channel electric field and blood glucose detection ability are well correlated.

## 2. Experimental Section

**Figure 1.**
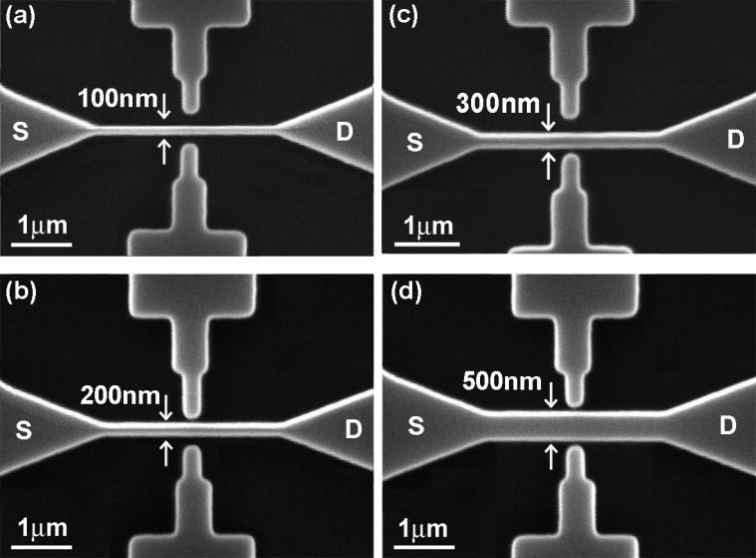
SEM images of the PSW sensors with channel widths of **(a)** 100 nm, **(b)** 200 nm, **(c)** 300 nm, and **(d)** 500 nm, respectively.

The PSW sensors were fabricated on a 12 nm-thick SiO_2_ coated p-type (100) silicon wafer. The polysilicon layer was doped with phosphorous and had a thickness of 80 nm and a sheet resistance of 40–50 Ω/□. The PSWs were patterned with different line widths by e-beam lithography. Following the development process, the PSWs were then lifted by reactive-ion etching. Figure 1(a–d) shows the SEM images of the PSWs with line widths of 100 nm, 200 nm, 300 nm and 500 nm respectively, and a channel length of 3 μm.

**Figure 2.**
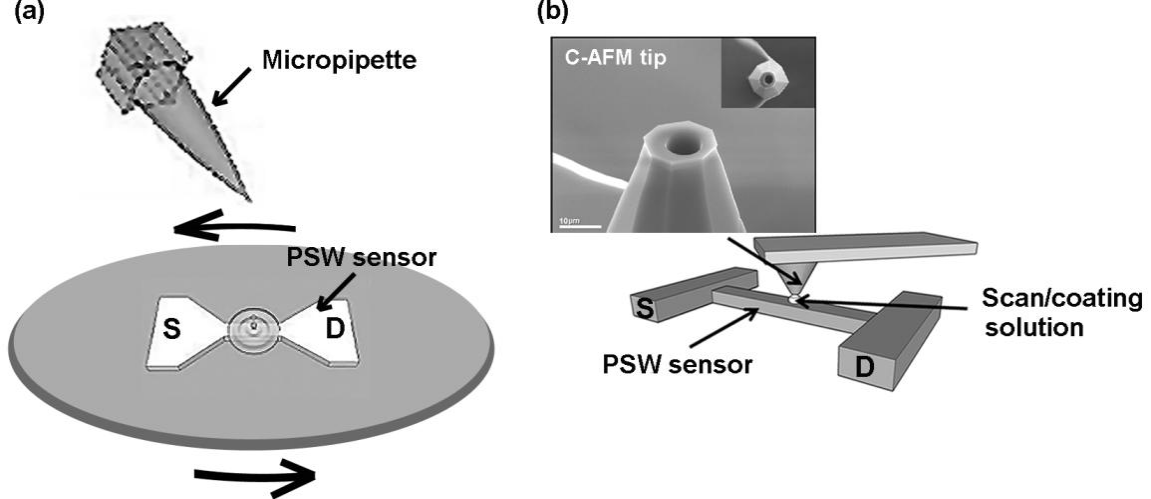
Schematic diagrams of the solution coating processes for **(a)** micropipette/spin-coating, and **(b)** FIB-processed C-AFM tip scan/coating. The SEM picture is of the FIB processed C-AFM tip.

Pure γ-APTES was first diluted in an ethanol solution with a mixed volume ratio of ethanol and pure γ-APTES of 100:1. The γ-APTES+NPs nanocomposite was composed of the diluted γ-APTES and PDMS-treated hydrophobic fumed silica NPs (R202, EVONIC industries). The mixed weight ratio of γ-APTES and NPs was 100:1 and the average primary silica particle size was 14 nm. The mixture was subjected to ultrasonic vibration for 10 minutes to disperse the silica NPs. Two methods were used for coating the sensing membrane: spin-coating and FIB milled C-AFM tip coating. Prior to the spin-coating process, the diluted γ-APTES or γ-APTES+NPs was loaded on a micropipette and dropped onto the PSWs with 1 μL for each device. The spin-coating process was performed at 3,000 rpm for 30 s. Details on the fabrication of the FIB milled C-AFM tip coating can be found in our previous work [8]. Via the AFM controller, the FIB processed C-AFM tip can load and transfer the solution to any pre-defined position on the sample surface. Prior to coating the γ-APTES or γ-APTES+NPs solution onto the sensor surface, the sample was first scanned by the AFM in non-contact mode at a resonance frequency of 14 kHz, a force constant of 0.2 N/m and a scan speed of about 1 Hz to confirm the PSW position. Then, the C-AFM tip was lifted and the mixture of the γ-APTES or γ-APTES+NPs solution was loaded into the cylindrical well of the tip using a micropipette without removing the sample. From the volume of the cylindrical well, the amount of the γ-APTES or γ-APTES+NPs solution deposited was estimated to be ∼3 × 10^−2^ pL. The C-AFM tip was then placed onto the PSW surface with a contact force of 1 nN. An area of 3 μm × 3 μm was scanned so that the solution was coated over the entire scanned region at the same time. Figures 2(a,b) shows the schematic diagrams for the micropipette/spin-coating and FIB processed C-AFM tip scan/coating methods [8]. Following the coating of the γ-APTES or γ-APTES+NPs layer, the samples were cured on a hotplate at 120 °C for 5 min. Since it is believed that UV exposure can enhance the covalent bond strength between NH_2_ molecules and silica NPs [20,21], following curing some of the samples were illuminated with UV light (wavelength = 365 nm) at different exposure times. For the sake of comparison, the rest of the samples were not exposed to UV.

Thirty mg of glucose oxidase (GOx) (EC.1.1.3.4, Sigma) was dissolved into a phosphate buffer solution mixed with 20 μL β-d-glucose solution with different concentrations under test. For the PSWs with spin-coated membranes, the mixtures of the β-d-glucose solution and GOx were dropped by micropipette. For the PSWs with C-AFM coated membranes, the mixtures of the β-d-glucose solution and GOx were coated onto the PSW surface with the FIB processed C-AFM tip, as in the γ-APTES or γ-APTES+NPs mixture scan/coating process. After dropping or coating the glucose solutions under test onto the PSW surfaces, a semiconductor parameter analyzer Agilent 4156C was used to measure the currents flowing through the PSW channels. The PSW channel current changes before and after applying the glucose solution, Δ*I* = *I*(after coating) – *I*(before coating), were then determined. The AFM system used in this work was the SEIKO 300 HV.

## 3. Results and Discussion

### 3.1. Coating Thickness Analysis for the γ-APTES+NPs Nanocomposite Membrane

**Figure 3.**
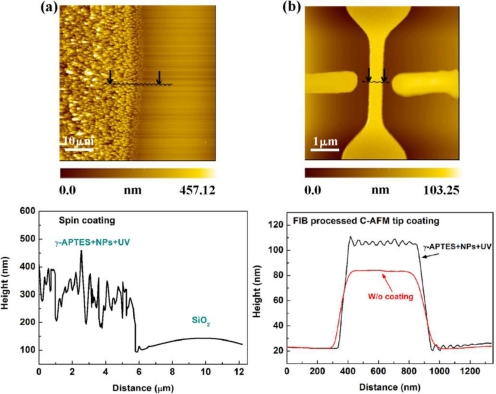
AFM pictures and surface profiles for the PSW coated with γ-APTES+NPs membrane using **(a)** micropipette/spin-coating, and **(b)** FIB processed C-AFM tip scan/coating.

The thickness of the coated γ-APTES+NPs nanocomposite was determined by the cross sectional surface profile of the membrane measured by the AFM system. Because the spread area of the γ-APTES+NPs nanocomposite is much larger than the maximum area that can be scanned by the AFM (60 μm × 60 μm), the thickness of the micropipette/spin-coated membrane was determined by the surface profile at the side edge of the membrane. Because the γ-APTES+NPs nanocomposite is localized at the PSW, the thickness of the C-AFM tip scan/coated membrane could be determined by the surface profile at the wire region. Figure 3(a) shows the AFM picture at the edge of the spin coated γ-APTES+NPs and its corresponding cross sectional surface profile. The AFM image shows that the thickness of the spin coated γ-APTES+NPs varies from about 450 to 470 nm. Since it is difficult to obtain a smooth surface profile for a membrane with large thickness variation by AFM, we used the peak value of the surface profile as the thickness of the membrane. Figure 3(b) shows the AFM image of the PSW with C-AFM tip coated γ-APTES+NPs and its corresponding cross sectional surface profile. The surface profile of the PSW without coating γ-APTES+NPs is also shown for comparison. It is found that a smooth profile can be obtained for the C-AFM tip coated membrane with a thickness of about 21–23 nm.

**Figure 4.**
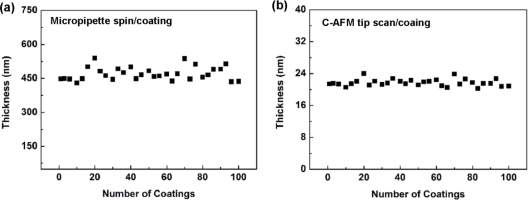
Thickness variations of the γ-APTES+NPs membranes for one hundred repeated **(a)** micropipette/spin-coating, and **(b)** FIB processed C-AFM tip-coating, respectively.

Figure 4(a,b) shows the thickness variations of the γ-APTES+NPs membranes for one hundred repetitions of spin-coating and C-AFM tip-coating, respectively. The RSD of the 100 thicknesses for spin-coating and C-AFM tip-coating are 12.76% and 5.33%, respectively.

**Figure 5.**
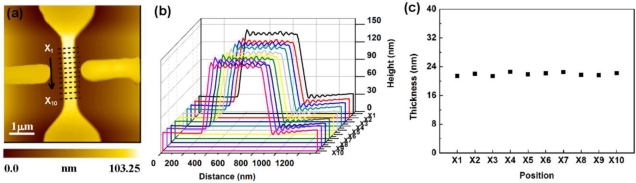
Membrane thickness variations for different positions on a single PSW using FIB processed C-AFM tip scan/coating.

We also checked the membrane thickness variation for different positions on a single PSW. The results are shown in Figure 5. The RSD of the 10 thicknesses for different positions marked in Figure 5(a) is 5.483%. As observed from Figures 4 and 5, we can uniformly and repeatedly coat an ultra-thin γ-APTES+NPs nanocomposite membrane onto the PSW.

### 3.2. Characteristics of Sensitivity Performance of the PSW Sensor

**Figure 6.**
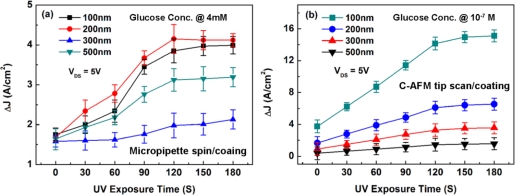
Current density changes ΔJ as a function of UV exposure time for PSWs with different line widths coated with γ-APTES+NPs membrane by **(a)** micropipette/spin-coating, and **(b)** C-AFM tip scan/coating.

This section examines the line width dependence, UV exposure time and spin-coating/tip-coating effects on the sensitivity characteristics of the PSW. Figure 6(a,b) shows the effect of UV exposure time on the ΔJ measured at a drain-to-source voltage V_DS_ = 5 V for different channel-wide PSWs coated with a γ-APTES+NPs membrane layer. The addition of NPs results in the γ-APTES+NPs offering a substantial sensing surface area. The surface-charge state is altered by the binding of hydrogen ions with the NH_2_ bonds of γ-APTES, changing the conductivity of the PSW channel, and correspondingly changing the current flowing through the PSW channel. The tested glucose concentration for spin-coated and tip-coated membranes was set at 4 mM and 10^−7^ M, respectively. Figure 6(a) shows that, for the PSW coated with spin-coated γ-APTES+NPs, the ΔJ increases with UV exposure time for the first 90 s, and begins to saturate after 120 s for the 100 nm-, 200 nm-, 300 nm- and 500 nm-wide PSWs. However, there is random relationship between the sensitivity and channel width but Figure 6(b) shows that, for the PSW coated with C-AFM tip-coated γ-APTES+NPs, the ΔJ increases almost linearly with UV exposure time for the first 90 s, and begins to saturate after 120 s for PSWs of all different widths.

In addition, the ΔJ shows a strong correlation with the line width. The PSW with a narrow line width exhibits high sensitivity at the same UV exposure time. The increase of ΔJ with UV exposure time is believed to be due to the increasing surface roughness following UV illumination [8]. Hydrogen peroxide is produced from the glucose catalysis by the GOx, at which point the electrical field electrolyzes the H_2_O_2_ to generate hydrogen ions. As mentioned earlier, the H^+^ ions can bind with NH_2_ bonds of γ-APTES+NPs, changing the surface-charge state of the PSW and correspondingly changing the current flowing through the channel. The catalytic and electrolysis reactions are expressed in Equations (1) and (2):

(1) $$\text{glucose}+O_{2}\overset{{\text{GO}}_{x}}{\to }\text{gluconic }\;\;\;\text{acid}+H_{2}O_{2}$$

(2) $$H_{2}O_{2}\overset{\text{Electricalfield}}{\to }O_{2}+{2H}^{+}+{2e}^{-}$$

**Figure 7.**
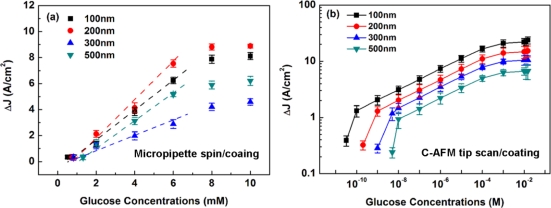
Current density changes ΔJ as a function of glucose concentrations for PSWs with different line widths coated with γ-APTES+NPs+UV by **(a)** micropipette/spin-coating, and **(b)** C-AFM tip scan/coating.

Figure 7(a) shows the line width dependence of the channel current density changes ΔJ as a function of glucose concentrations for the PSW spin-coated with γ-APTES+NPs plus 90 s of UV illumination. It was found that the linear region extended from 1 mM to 6 mM for all line widths, and the lowest detection limits were 0.75, 0.82, 0.52 and 1.32 mM for the 100 nm-, 200 nm-, 300 nm- and 500 nm-wide PSWs, respectively. The order of the ΔJ (*i.e.*, the order of sensitivity) is ΔJ(200 nm) > ΔJ(100 nm) > ΔJ(500 nm) > ΔJ(300 nm). It is noted that, for the spin-coated membranes, the lowest detection limits and the ΔJs exhibited a random relationship between different line widths of the PSW sensors. Figure 7(b) shows the line width dependence of the channel current density changes ΔJ as a function of glucose concentrations for the PSW tip-coated with γ-APTES+NPs plus 90 s of UV illumination. It was found that the linear regions for 100 nm-, 200 nm-, 300 nm- and 500 nm-wide PSWs were 0.1 nM to 1 mM, 1 nM to 1 mM, 5 nM to 1 mM, 10 nM to 1 mM, respectively. The lowest detection limits were 32 pM, 0.2 nM, 1 nM and 5 nM, for the 100 nm-, 200 nm-, 300 nm- and 500 nm-wide PSWs, respectively. As observed, for the PSW coated with γ-APTES+NPs+UV by the FIB-processed C-AFM tip, the linear region and ΔJ increased as line width decreased. The lowest detection limit decreased with line width. The order of the ΔJ (*i.e.*, the order of sensitivity) is ΔJ(100 nm) > ΔJ(200 nm) > ΔJ(300 nm) > ΔJ(500 nm). The PSW with tip-coating processes showed good correlation with line width in terms of sensitivity characteristics. The ultra-thin γ-APTES+NPs coated by C-AFM tip and the introduction of NPs that caused the highly sensitive for PSW.

**Figure 8.**
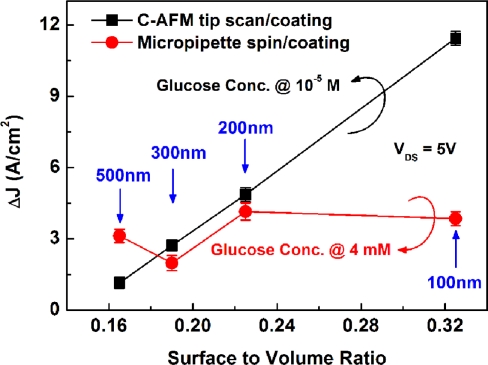
Channel current density changes ΔJ as a function of surface-to-volume ratio for PSWs with different line widths coated with γ-APTES+NPs+UV by micropipette/spin-coating and C-AFM tip scan/coating.

Figure 8 illustrates the ΔJ as a function of surface-to-volume ratio calculated from Figure 7(a,b) for different line widths. Here, the glucose concentrations chosen for illustration were 1 × 10^−5^ M and 4 × 10^−3^ M respectively, located at the middle points of the linear region in Figure 7(a,b).

Figure 8 shows that, the ΔJ has an excellent linear relationship with the surface-to-volume ratio of the PSW with the tip-coating membrane, but shows an irregular relationship for the PSW with the spin-coating membrane. Since the PSWs were coated individually in the tip-coating process, it is believed that ultra-thin and uniform γ-APTES+NPs nanocomposite membranes could be coated repeatedly with the FIB milled C-AFM tip. This is consistent with the thickness analysis in the above section.

**Figure 9.**
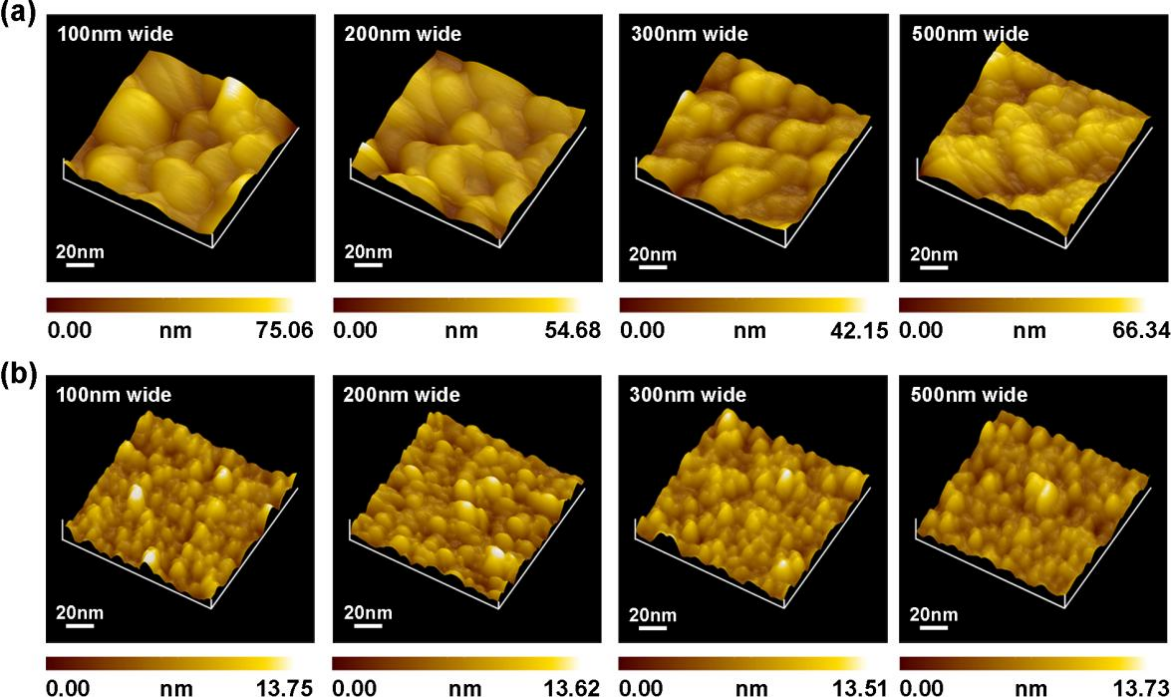
AFM images of the surface morphology for the γ-APTES+NPs coated onto 100 nm-, 200 nm-, 300 nm-, 500 nm-wide PSW surface with UV illumination 120 s by **(a)** micropipette/spin-coating, and **(b)** C-AFM tip scan/coating.

To further investigate the difference of line width dependence on the sensitivity between spin-coating and tip-coating membranes, Figure 9(a,b) respectively shows the AFM surface morphology pictures of the 100 nm-, 200 nm-, 300 nm- and 500 nm-wide PSW coated with a γ-APTES+NPs nanocomposite plus 120 s of UV illumination by spin-coating and tip-coating. For the spin-coated membrane, Figure 9(a) shows that large aggregated grains formed in the membrane. The large and varied grain sizes result in the irregular relationship between the ΔJ and the surface-to-volume ratio of PSWs in Figure 8. As for the tip-coating membrane, Figure 9(b) shows many small clusters uniformly distributed on the PSW surface. The range of the root-mean-square (RMS) of the surface roughness of the γ-APTES+NPs+UV nanocomposite-modified PSWs with different channel widths is about 1.67 nm to 1.75 nm. Since the tip-coating membrane is about 22 nm-thick, and the average size of the silica NPs is 14 nm, we can coat a uniform membrane with only one to two layers of silica NPs by the C-AFM tip. The ultra-thin membrane and uniformly-distributed clustered surface caused the γ-APTES+NPs+UV nanocomposite-modified PSW to exhibit ultra-high sensitivity and an excellent linear relationship between ΔJ and the surface-to-volume ratio.

### 3.3. Characteristics of Selectivity Performance of the PSW Sensor

Glucose sensors are commonly used in the presence of different kinds of potential interferences, and it is imperative that the glucose sensor be immune to any such interference during the sensing operation. In our recent work [10], we demonstrated that the PSW with γ-APTES+NPs+UV nanocomposite-modified surface was highly immune to interference. Because there is no need for electrolysis electrodes in the PSW sensor, and the γ-APTES+NPs+UV nanocomposite exhibits low leakage characteristics, the percentage change of the PSW channel current changes ΔI was as low as 10% at an ultra-high acetaminophen (AP)-to-glucose concentration ratio of 600:1. In this work, we further investigate the interference-immunity of PSW glucose sensors with different line widths. Table 1 illustrates the percentage changes of ΔJ under various AP-to-glucose concentration ratios for different membranes. The AP-to-glucose concentration ratios range from 0.003:1 to 600:1 with a fixed glucose concentration of 1 × 10^−5^ M, and ΔJ measured at V_DS_ = 5 V. It is found that, for the whole range of AP-to-glucose concentration ratios, the percentage changes of ΔJ corresponding to the PSW tip-coated with γ-APTES+NPs and γ-APTES+NPs+UV are much lower than those for the PSW tip-coated with γ-APTES [10]. The percentage changes of ΔJ were as follows: ΔJ (100 nm) > ΔJ (200 nm) > ΔJ (300 nm) > ΔJ (500 nm) for all the PSWs tip-coated with different membranes. The disturbance caused by AP interference in the γ-APTES+NPs+UV-modified 500 nm line-wide PSW can be kept below 3.5% of the original sensing current density for an AP-to-glucose concentration ratio up to 600:1. The line width dependence of interference immunity is mainly attributed to the different fringing electrical fields induced in the sensing membrane which were strongly correlated with the channel electrical field in the PSW.

To understand the channel width dependence of the electrical field in PSWs, numerical simulations of channel electrical fields for difference channel widths were carried out using MATLAB. The Fermi level pinning and other surface effects were neglected and the PSWs were assumed to be fully passive. The source to drain current I_DS_ at V_DS_ = 5 V for 100 nm-, 200 nm-, 300 nm- and 500 nm-wide PSWs follows the expression:

(3) $$I_{\text{DS}}=\frac{{q\mu }_{n}}{L}\int_{A}n(V)\text{dA}$$

where *q* is the electron charge, *μ_n_* is the electron mobility of about 1,000 cm^2^ Vs^−1^, *L* is the PSW length of 3 μm, and A is the cross section area of the PSW. The electron concentration *n(V)* at source to drain bias V_DS_ = 5 V can be obtained by Equation (3). The channel electric potential *V* of the PSW can be solved from Equations (4) and (5) which are derived from the Poisson’s equation according to device geometries:

(4) −∇⋅[ɛ∇V]=ρ

(5) $$\rho =q\cdot [N_{d}-n(V)+p(V)]\approx q\cdot [N_{d}-n(V)]$$

where the space charge density ρ = θ · [N_δ_ – *v*(ς)] is for n-type PSW, ɛ = ɛ_0_ɛ_r_ is the dielectric constant for polysilicon of about 11.9, *N_d_* is the PSW’s doping concentration of about 5 × 10^14^ cm^−3^. The electrical potential was solved by Poisson’s equation with the Neumann boundary condition, and the channel electrical field strength of the n-type PSW can be obtained by:

(6) E=−∇V

**Figure 10.**
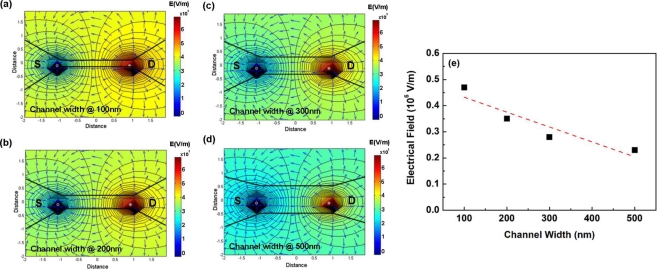
Channel electrical field simulation for the PSWs with channel widths of **(a)** 100 nm, **(b)** 200 nm, **(c)** 300 nm, and **(d)** 500 nm. **(e)** is the linear fitting of the electrical field strength for the corresponding simulation.

Figure 10 shows the channel electrical field strength simulation results for the (a) 100 nm-, (b) 200 nm-, (c) 300 nm- and (d) 500 nm-wide PSWs under a drain-to-source voltage V_DS_ of 5 V. The channel electrical field strength for the 100 nm-, 200 nm-, 300 nm- and 500 nm-wide PSWs is about 0.47 MV/m, 0.35 MV/m, 0.28 MV/m and 0.23 MV/m, respectively, as plotted in Figure 10(e).

The order of the channel electrical field intensity in the PSW is as follows: E(100 nm) > E(200 nm) > E(300 nm) > E(500 nm). Thus, the electrical field strength decreases with PSW line width, which is consistent with the results shown in Table 1. For a PSW with a wider line width, the smaller channel electrical field was expected to induce a weak fringing electrical field in the sensing membrane. Therefore, the interferent could not be electrolyzed to generate a disturbing signal in the glucose detection, thus leaving the 500 nm-wide PSW highly immune to interference.

## 4. Conclusions

We can use the FIB-milled C-AFM tip-coating process to repeatedly apply an ultra-thin γ-APTES+NPs nanocomposite layer with uniform thickness and well-distributed NPs on the PSW, resulting in a good linearity in ΔJ *versus* the surface-to-volume ratio plot. The membrane thickness RSD for 100 repeated micropipette/spin-coatings and C-AFM tip scan/coatings were 12.76% and 5.33%, respectively. The sensitivity was strongly correlated with the line width for the PSW with the tip-coating membrane, but showed randomly ordered with the line width for the spin-coated PSW. The PSW glucose sensors with smaller channel widths exhibit a higher sensitivity only with the tip-coating process. PSWs with a wider line width had a smaller channel electrical field and were expected to induce smaller fringing electrical fields in the membrane, resulting in better immunity to interference.

## Figures and Tables

**Table 1.** Percentage changes of ΔJ (%) measured at V_DS_ = 5 V for PSWs with different line widths, tip-coated with γ-APTES, γ-APTES+NPs and γ-APTES+NPs+UV layers, respectively. The glucose detections were performed under various AP-to-glucose concentration ratios.

**Membranes**	**NW channel widths (nm)**	**Acetaminophen/glucose molar ratio**							

		**0.003:1**	**0.01:1**	**0.1:1**	**1:1**	**10:1**	**100:1**	**300:1**	**600:1**
**γ-APTES**	100	–	9.1	9.12	12	17	22.1	27.4	33.5
	200	–	8	8.4	10	13	17	21.3	25.7
	300	–	4.5	6.3	6.5	8	10	12	15
	500	–	4	5	5.7	5.8	5.9	8.3	10.5

**γ-APTES+NPs**	100	–	6.3	8.2	8.31	8.4	8.7	9.65	11.24
	200	–	5.2	5.8	6.1	6.15	6.3	7.4	8.32
	300	–	4	4.7	4.9	5.4	5.43	6.22	8
	500	–	3.7	4.1	4.3	4.4	4.51	5.3	6.8

**γ-APTES+NPs+UV**	100	–	4.5	5.1	5.6	6.7	7	8.5	9.7
	200	–	3.2	3.3	3.4	3.4	3.8	5	6.1
	300	–	2.5	3	3.3	3.3	3.3	4.1	4.8
	500	–	1	2.2	2.2	2.2	2.3	3.2	3.5

(−) No changes
